# Selective aortic arch perfusion: a first-in-human observational cadaveric study

**DOI:** 10.1186/s13049-023-01148-z

**Published:** 2023-12-12

**Authors:** Max Marsden, Jon Barratt, Helen Donald-Simpson, Tracey Wilkinson, Jim Manning, Paul Rees

**Affiliations:** 1 Blizard Institute, The Faculty of Medicine and Dentistry, Queen Mary University of London, 4 Newark Street, London, E1 2AT UK; 2 Defence Endovascular Resuscitation Group, Research and Clinical Innovation, Birmingham, UK; 3 East Anglian Air Ambulance, Helimed House, Norwich, UK; 4 Tayside Innovation MedTech Ecosystem TIME, University of Dundee, Wilson House, Dundee, DD2 1FD UK; 5 Human Anatomy Unit, College of Medical and Dental Sciences, University of Birmingham, Birmingham, UK; 6 Department of Emergency Medicine, University of North Carolina at Chapel Hill, Chapel Hill, USA

**Keywords:** Resuscitation, Endovascular, Trauma, Cardiac arrest, SAAP

## Abstract

**Background:**

Selective aortic arch perfusion (SAAP) is a novel endovascular technique that combines thoracic aortic occlusion with extracorporeal perfusion of the brain and heart. SAAP may have a role in both haemorrhagic shock and in cardiac arrest due to coronary ischaemia. Despite promising animal studies, no data is available that describes SAAP in humans. The primary aim of this study was to assess the feasibility of selective aortic arch perfusion in humans. The secondary aim of the study was to assess the feasibility of achieving direct coronary artery access via the SAAP catheter as a potential conduit for salvage percutaneous coronary intervention.

**Methods:**

Using perfused human cadavers, a prototype SAAP catheter was inserted into the descending aorta under fluoroscopic guidance via a standard femoral percutaneous access device. The catheter balloon was inflated and the aortic arch perfused with radio-opaque contrast. The coronary arteries were cannulated through the SAAP catheter.

**Results:**

The procedure was conducted four times. During the first two trials the SAAP catheter was passed rapidly and without incident to the intended descending aortic landing zone and aortic arch perfusion was successfully delivered via the device. The SAAP catheter balloon failed on the third trial. On the fourth trial the left coronary system was cannulated using a 5Fr coronary guiding catheter through the central SAAP catheter lumen.

**Conclusions:**

For the first time using a perfused cadaveric model we have demonstrated that a SAAP catheter can be easily and safely inserted and SAAP can be achieved using conventional endovascular techniques. The SAAP catheter allowed successful access to the proximal aorta and permitted retrograde perfusion of the coronary and cerebral circulation.

**Supplementary Information:**

The online version contains supplementary material available at 10.1186/s13049-023-01148-z.

## Background

Haemorrhage remains the leading cause of preventable death after injury in both military and civilian casualties [1, 2]. Whilst evidence suggests that overall patient outcomes have improved following battlefield injury [3], patients with non-compressible torso haemorrhage have not demonstrated improved outcomes [4, 5]. It is imperative that novel technologies are introduced to improve patient outcome in high risk patients, such as those with non-compressible torso haemorrhage [6, 7]. Current endovascular therapies such as Resuscitative Endovascular Balloon Occlusion of the Aorta (REBOA) show promise as temporary haemorrhage control tools. However, REBOA is not currently supported by robust evidence from randomised control trials and may in some settings cause harm [8, 9]. In situations of established cardiovascular collapse pure aortic occlusion might not be sufficient to enable a return of spontaneous circulation [10–12].

**Fig. 1:**
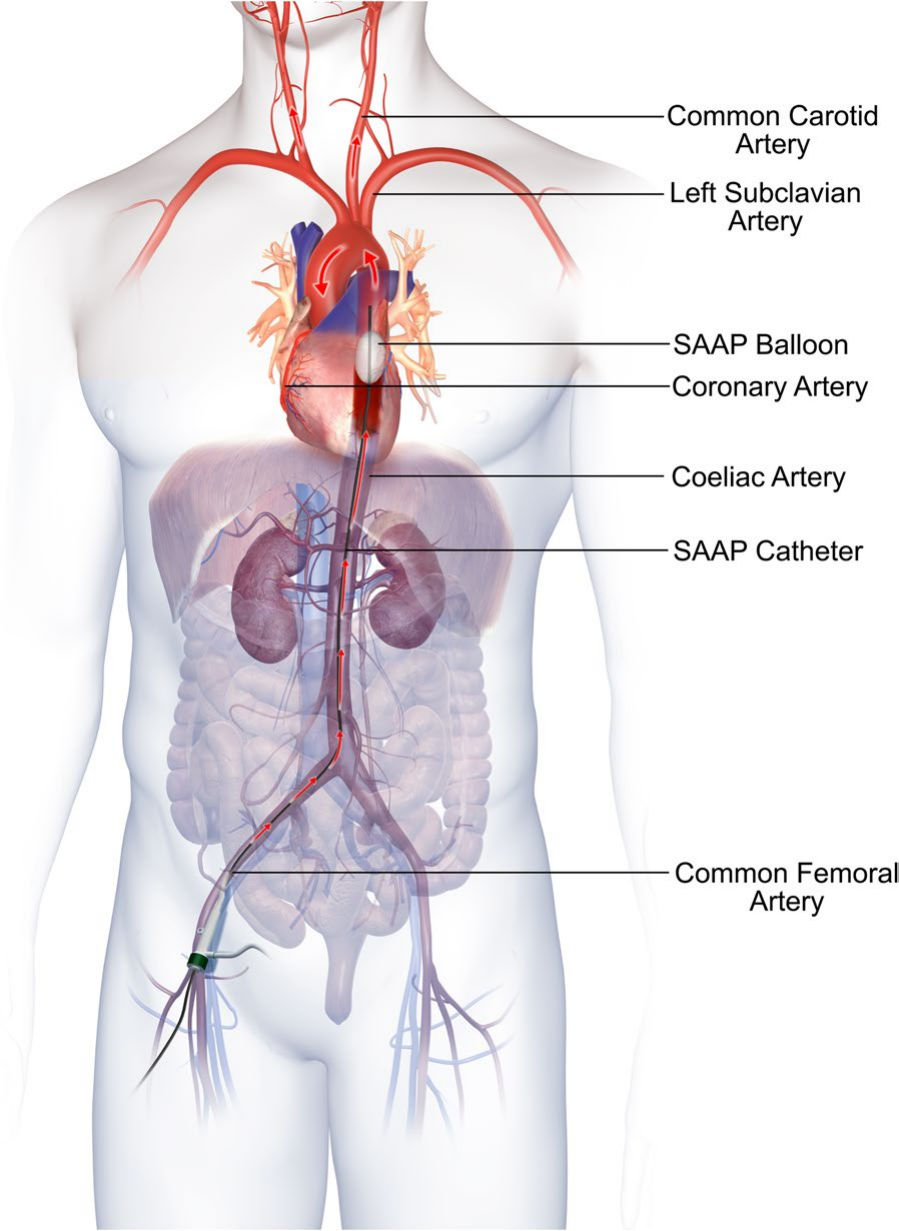
Selective Aortic Arch Perfusion (SAAP) catheter placement in humans

Selective aortic arch perfusion (SAAP) is an endovascular technique that combines thoracic aortic occlusion with extracorporeal perfusion of the brain and heart (Fig. 1) [13]. SAAP is associated with improved survival in animal models of haemorrhage induced traumatic cardiac arrest compared to REBOA. SAAP has been shown to improve survival in traumatic cardiac compared to REBOA in animal models of haemorrhagic cardiac arrest [14]. SAAP involves placing a balloon occlusion catheter into the descending aorta through the common femoral artery. The SAAP catheter provides a means to establish retrograde perfusion of the aortic arch through a central lumen.

Apart from its role in haemorrhagic shock, SAAP could also be used in medical cardiac arrest and critical hypotension due to coronary ischaemia. To facilitate emergency percutaneous coronary intervention (PCI) for myocardial infarction, SAAP could be employed when managing a patient in a peri-arrest or critically shocked state. In this context augmented perfusion of the aortic arch during PCI would enhance cerebral and coronary perfusion simultaneously. Here SAAP might be carried out as a preliminary step or an alternative to full mechanical cardiovascular support, such as veno-arterial extracorporeal membrane oxygenation (VA-ECMO).

Despite promising animal studies of SAAP [12, 14, 15], no feasibility data exist to support its use in humans. In addition, no SAAP device currently has regulatory body approval for human use. The primary aim of this study was to assess the feasibility of SAAP in humans. The secondary aim was to assess the feasibility of achieving direct coronary artery access via the SAAP catheter as a potential conduit for salvage PCI.

## Methods

**Fig. 2:**
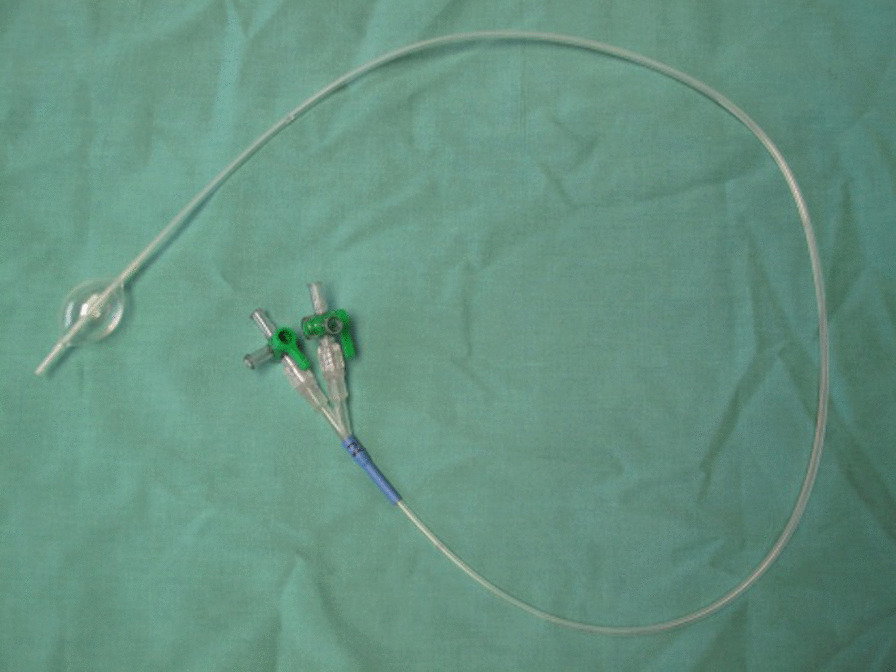
Experimental SAAP catheter prototype (Vention Medical Inc, USA). Catheter is 800mm long, 11.5Fr outer diameter, 7.5Fr central lumen, with a 30mm (17 ml) semi-compliant balloon near the tip that is inflated via a separate lumen

This observational study was performed in a university laboratory environment using Thiel embalmed human cadavers. The study was conducted by an interventional cardiologist (PR) familiar with intra-arterial access and fluoroscopic imaging. The SAAP catheter was an experimental catheter prototype that was 800mm long with an 11.5 Fr outer diameter (Vention Medical Inc, USA). It had a 7.5 Fr central lumen, and a 30 mm (17 ml) semi-compliant balloon near the tip that was inflated via a separate lumen. (Fig. 2) Multiple catheters were used throughout the study. The study was performed four times, with the same SAAP catheter used in each trial.

### Cadaveric aortic flow and SAAP cannulation

Two cadavers were preserved using the Thiel technique [16, 17]. A previously validated method to establish extracorporeal driven ante-grade aortic flow within the cadaver was adapted to deliver extracorporeal flow retaining the Thiel cadaver completely intact without chest plate removal [18]. This provided anatomically accurate, physiologically similar and robust model comparable to a model previously used to simulate aortic endovascular procedures [18]. Ports (16 Fr x 10cm Check-Flo, Cook Medical, USA) were placed into the cadaver’s left and right subclavian arteries and connected to a cardiopulmonary bypass machine (HL30 Maquet, Germany). A modified master pump recreated pulsatile flow at 60 beats per minute. The master pump was prepared with 6m of 13mm diameter silicone-platinum coated tubing (Silex, UK), delivering a flow rate of approximately 2.5L per minute as the standard setting. Two 14 Fr vascular access sheaths (Medtronic, UK) fitted with a haemostatic valve adapter were placed in the left and right common femoral artery using an open surgical cutdown technique and sutured in place. Through this, the SAAP catheter (Vention Medical Inc, USA) was introduced over a 180cm, J-tipped 0.035″ PTFE-coated wire (Amplatz super-stiff, Boston Scientific, USA). Pressure transducers (TruWave, Edwards Lifesciences, USA) were connected to a haemodynamic monitoring system (M3046A, HP, Germany) to provide a continuous pressure measurement at three separate sites: extracorporeal circuit, descending thoracic aorta and abdominal aorta.

### Positioning of the SAAP Catheter and Balloon Inflation

Once pulsatile flow had been established in the cadaver’s central vasculature the SAAP device was inserted via the right common femoral artery. Appropriate positioning of the SAAP catheter was achieved using fluoroscopic guidance (OEC Elite 9900 C-arm, GE, USA) at 8–12 frames per second (fps), and digital subtraction angiography at 25 fps. The balloon end of the SAAP device was positioned in the descending aorta, distal to the left subclavian artery. Once in position, the balloon was inflated under live fluoroscopic guidance. A 50:50 mix of normal saline and iodinated contrast medium (Omnipaque 300, GE Healthcare, Auckland) was used to inflate the balloon. Aortic apposition was confirmed radiologically, by tactile assessment, and by changes in the extracorporeal flow and pressure indications.

### Aortic arch perfusion and direct coronary artery access

Following successful inflation of the SAAP balloon in the descending aorta, a 30ml bolus of 50:50 saline-contrast mix was delivered through the central lumen of the SAAP catheter to the aortic arch via an autoinjector. Cine-fluoroscopy was continued during this process, to assess whether coronary flow was achieved. To assess the feasibility of achieving direct coronary access via the SAAP catheter a Judkins left 4.0 5Fr coronary guiding catheter (Vista Brite Tip, Cordis, UK) was placed through the SAAP catheter. This was performed after the SAAP balloon was inflated in the thoracic aorta. Using standard techniques engagement of the left main stem was attempted, confirmed with hand-injection of contrast via a conventional syringe-manifold.

### Identifying procedure success and complications

Systematic post hoc review of the images was performed to identify correct device placement, overt aortic injury, subtle intimal damage and dissection, ability to deliver bolus to aortic root, balloon patency, aortic valve position, positive carotid, and coronary artery flow.

### Ethical considerations

Study procedures involving human cadaveric participants were carried out in accordance with current anatomical legislation, the Anatomy Act 1984 and Human Tissue (Scotland) Act 2006.

## Results

**Fig. 3:**
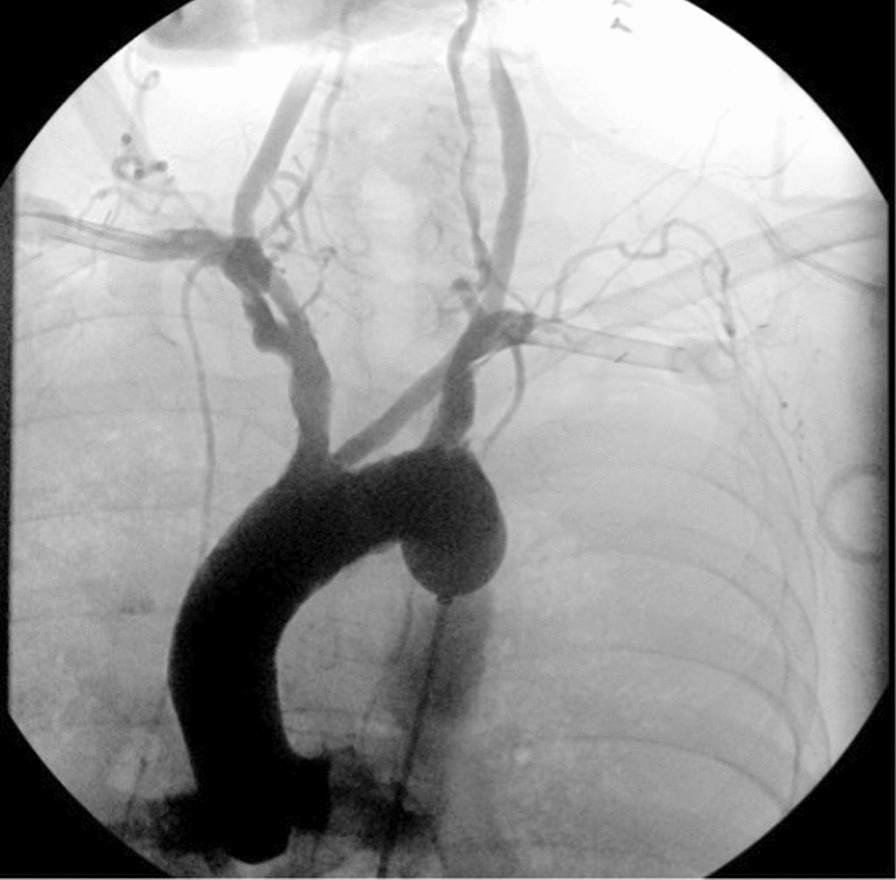
Fluoroscopic image with digital subtraction angiography enhancement demonstrating selective aortic arch perfusion of contrast. The SAAP catheter is visualised in the descending aorta. The aortic arch and its branches are opacified. The left and right subclavian arteries have been canulated to establish extracorporeal pulsatile flow

During the first and second attempts at SAAP the device was passed rapidly and without incident to the intended descending aortic landing zone, visualised with fluoroscopy. Full balloon apposition was confirmed, and in all cases aortic arch perfusion was successfully delivered via the device. (Fig. 3) Minor cephalad migration of the device was noted during run 2, possibly caused by catheter movement during connection to the SAAP autoinjector. No evidence of aortic injury was noted on careful post hoc evaluation of the images. In both SAAP cases, full opacification of the proximal aorta was seen, with opacification of both carotid and coronary systems, confirming the possibility of initiating successful human SAAP through this device. (Additional file 1).

In a third run, SAAP was not possible due to a failure of sustained balloon inflation. After delivery to the correct anatomical landing zone, balloon rupture was detected which prevented balloon apposition, with translocation of circulating fluid into the balloon inflation device. Following removal of the SAAP device, examination identified a defect close to a seal-zone on the trailing edge of the balloon.

**Fig. 4:**
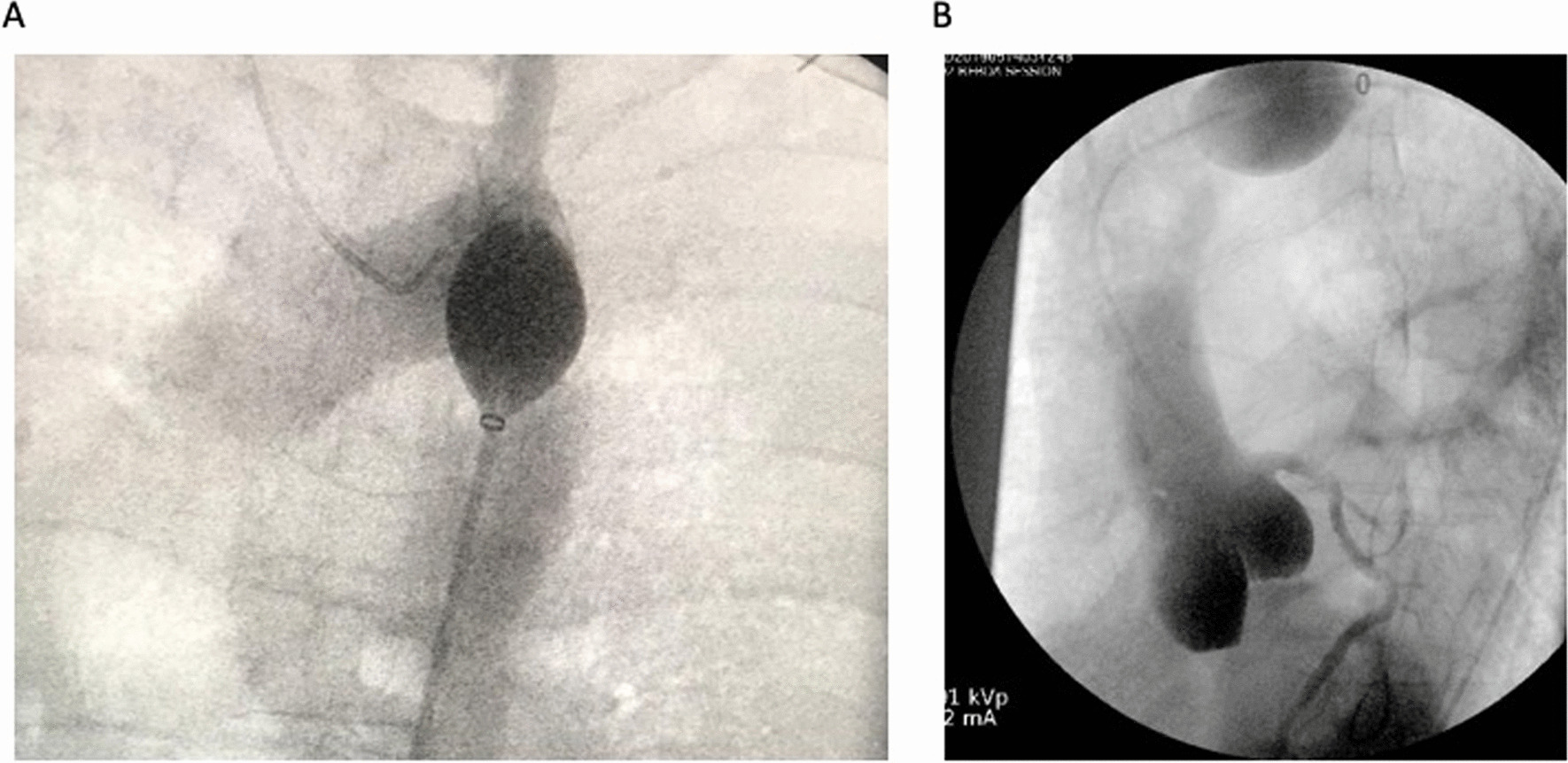
Selective aortic arch perfusion (SAAP) catheter in the aorta with Judkins left 4.0 catheter via the SAAP central lumen engaged in the left main stem. **A** without contrast. **B** with contrast in the left coronary artery

To assess the feasibility of achieving direct coronary access via the SAAP catheter, a fourth trial run was performed, with a new SAAP catheter. Using the SAAP central lumen, it was possible, to access and engage the left coronary system using the 5 Fr coronary guiding catheter, and to opacify the left coronary systems. (Fig. 4).

## Discussion

For the first time, we have shown that the SAAP catheter, previously an animal-model laboratory research tool, can be inserted safely into a human using standard percutaneous endovascular techniques. Importantly, there was no evidence of immediate femoral, iliac or aortic damage. The ability to deliver aortic occlusion alongside concomitant retrograde aortic perfusion is a potentially useful prospect for the resuscitation of the most critically ill trauma patients. This would allow control of bleeding below the occlusion site, providing a blood-free field during damage control surgery, while simultaneously permitting the establishment of aortic arch extracorporeal circulation. The impact of such a device could save the fragile heart and brain, and potentially induce return of spontaneous circulation where hypovolaemic cardiac arrest has occured.

In addition to SAAP’s role in exsanguinating haemorrhage after subdiaphragmatic injury, the technique might have utility in the more common setting of critical hypotension from cardiogenic shock. SAAP has the potential to act as a bridge to salvage PCI or VA-ECMO. As we have shown in this study, the central lumen of the SAAP catheter can accommodate a coronary guide catheter to allow simultaneous aortic perfusion and emergency PCI through the same device.

It is useful to consider SAAP as a singular modality within a spectrum of endovascular techniques [10]. At one end of the spectrum is REBOA which provides aortic occlusion. The REBOA balloon can be inflated either in the thoracic aorta (Zone 1) or distal aorta (Zone 3). REBOA provides both haemorrhage control distal to the balloon and cardiac resuscitation proximal to it [19]. SAAP is procedurally more complex as it adds to the occlusion balloon of REBOA a central catheter through which fluids can be infused directly into the aortic arch. By having a central lumen, SAAP introduces an extracorporeal component, with flow rates up to 800 ml/min [14]. Further along the endovascular resuscitation spectrum are Extracorporeal Life Support (ECLS) through Extracorporeal Membrane Oxygenation (ECMO) and finally Emergency Preservation and Resuscitation (EPR). EPR represents a resuscitation approach in patients that have suffered a cardiac arrest from trauma [20]. Current EPR protocols perform rapid cooling to 10 °C through central canulation via thoracotomy. Once cooled there is a reduced risk of irreversible organ damage due to ischaemia and surgical haemostasis can be attempted. If the bleeding can be stopped the patient is then re-warmed on cardiopulmonary bypass [21].

Rapid cooling for EPR was initially achieved via an aortic balloon catheter inserted via the femoral artery to deliver an “aortic cold flush” into the aortic arch. Animal studies demonstrate that cooling delivered through a SAAP catheter can lead to good neurological outcomes in cardiac arrest [22, 23]. Such a strategy has some advantages as it may circumvent the need for thoracotomy to achieve central canulation. This minimally invasive approach maybe desirable in a pre-hospital phase of care for a patient in traumatic cardiac arrest.

There seems to be sufficient laboratory science to support moving toward a feasibility study in humans. From a purely technical standpoint SAAP can be implemented with current technologies. However, prior to embarking on clinical trials there are several areas to consider. First, there is a need to identify a safe and effective oxygen carrying perfusate. Many of the existing animal studies of SAAP support the use of fresh whole blood (FWB) as the perfusate [14]. As there are currently no licensed synthetic oxygen carriers autologous blood, with co-administration with calcium, appears to be the best option for the perfusate.

Second, which patients to include in a trial of SAAP also requires careful consideration. A trial in haemorrhage-induced cardiovascular collapse in trauma patients largely avoids concerns for volume overload and administering blood to medical cardiac arrest patients who are not bleeding. A trial of SAAP in trauma patients is likely to be complex to conduct. The recently published UK REBOA Trial has highlighted that patient selection, physician learning curves and delays to definitive care are challenges in the assessment of endovascular therapies [9]. Any planned feasibility study of SAAP in trauma patients must have strict inclusion and exclusion criteria. The study should be conducted in a setting where it's possible to safely and effectively gain access to the femoral artery, ideally before the patient experiences complete cardiovascular collapse. It's also critical that this system can simultaneously and expeditiously control severe bleeding. This would likely be a high volume centre with a proven track record of the highest quality trauma care.

This present cadaveric study has limitations. During the study there was one balloon rupture. This might have been caused either by either trauma to the balloon, perhaps due to repeated passage through the haemostatic valve adapter, or by primary seal failure. The experimental prototype SAAP catheter had been used in several previous experiments, and then transported internationally to allow this evaluation to take place. While no iatrogenic injuries were identified radiologically small injuries may not have been detected and the cadaveric preservation technique may have changed tissue behaviour. Another limitation of this study relates to initial placement guided by fluoroscopy. As an emergency resuscitation therapy *blind* SAAP catheter insertion, without the aid of fluoroscopy, is anticipated. This study did not assess a potential blind insertion technique in which insertion length is based on surface landmarks. It is possible that absolute control of balloon deployment site does not require close fluoroscopic control, as the physiological effect of SAAP extracorporeal perfusion appears to be similar with balloon placement anywhere between the left subclavian artery and the diaphragm [24] Finally, in this perfused cadaveric model we were not able to clearly visualise the right coronary arterial (RCA) system. This may be due to its anterior position and possible kinking of the vessels post-mortem, failure to achieve a coronary opening pressure sufficient to perfuse the RCA, or changes in transmyocardial impedance to flow after preservation. Alternative modelling techniques which remove the cadaver’s “chest plate” may improve access and visualisation of the RCA.

## Conclusions

We have demonstrated, for the first time in a perfused human cadaveric model, that a SAAP catheter can be effectively delivered via a standard femoral percutaneous access device. This delivery was apparently atraumatic, and easily achieved using conventional endovascular techniques. The SAAP catheter allowed successful access to the proximal aorta and permitted retrograde perfusion of the coronary and cerebral circulation.

### Supplementary Information

**Additional file 1**. Cine-fluoroscopy demonstrating selective aortic arch perfusion of contrast.

## Data Availability

Additional fluoroscopic images are available on request.

## Abbreviations

SAAP: Selective aortic arch perfusion
REBOA: Resuscitative endovascular balloon occlusion of the aorta
PCI: Percutaneous coronary intervention
VA-ECMO: Veno-arterial extracorporeal membrane oxygenation
